# The first reported case of Creutzfeldt‐Jakob disease from Nepal

**DOI:** 10.1002/ccr3.2609

**Published:** 2019-12-12

**Authors:** Himal Kharel, Pabitra Adhikari, Nishan B. Pokhrel, Zeni Kharel, Gaurav Nepal

**Affiliations:** ^1^ Tribhuvan University Institute of Medicine Kathmandu Nepal; ^2^ Department of Internal Medicine Rochester General Hospital Rochester NY USA

**Keywords:** Creutzfeldt‐Jakob disease, prion disorder, sporadic Creutzfeldt‐Jakob disease

## Abstract

Creutzfeldt‐Jakob disease (CJD) can also be diagnosed in a resource‐limited setting through good clinical analysis. The diagnosis of CJD should be considered in patients with rapidly evolving neurological signs associated with cognitive disturbances even in countries with limited available sophisticated tools and where CJD was never reported before.

## Graphical abstract

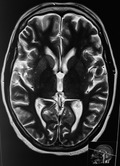

## 1 INTRODUCTION

We report a rare case of probable sporadic Creutzfeldt‐Jakob disease (CJD) in a 58‐year‐old lady who presented initially with treatment‐resistant depression. Imaging revealed subtle basal ganglia changes. The electroencephalogram showed periodic 1 Hz biphasic discharges typical of CJD. Supportive care was provided but her condition rapidly worsened.

Creutzfeldt‐Jakob disease (CJD) is a rapidly progressive, rare, transmissible, universally fatal, spongiform neurodegenerative condition caused by Prion protein.^1^ Normal cellular prion protein (PrP^C^) is found on cell membranes throughout the mammalian body. Disease‐causing form of Prion (PrP^Sc^) multiplies by binding to the normal cellular isoform PrP and converts it into an abnormal, structurally altered disease‐causing PrP^Sc^, which then spreads and accumulates throughout the brain leading to spongiform neurodegeneration.^1^ CJD can be present in any of four forms, namely sporadic (85%), genetic (10%‐15%), iatrogenic (<1%), and variant CJD (<1%).^2^ The average annual mortality rate, which also describes the incidence of this rapidly progressing disease has doubled from 1993 to 2018 (0.9 cases to 1.8 cases per million population, respectively).^3^ CJD has a long asymptomatic incubation period and a short symptomatic period with an invariably fatal outcome leading to death. Its initial diagnosis may be obscured by a variable presentation.

We present a case report that includes the clinical and radiological features of the first reported case of sporadic CJD (sCJD) in Nepal, and also illustrates the complexity of diagnosing this disease in the early stages of a clinical course in resource‐limited settings.

## 2 CASE REPORT

A 58‐year‐old nondiabetic normotensive lady visited our center with a chief complaint of abnormal behavior for 2 months. She was in perfect order 2 months ago, when she gradually began to feel the low mood, psychomotor slowdown, fatigue, decreased appetite, and anhedonia. It was not preceded by flu‐like illness or trauma. Her bowel and bladder habits were normal. She had no fever, headache, loss of vision, loss of consciousness, myalgia, arthralgia, tremor, sensory or motor seizures, or signs of hypothyroidism. There was no history of changes in sleep patterns, weight loss, malignancy, and exposure to toxic substances. Her professional history was not significant. She did not drink alcohol and did not smoke cigarettes. There was no history of drug abuse or prior immunosuppressive therapy. She had no recent infectious contacts. She was a nonvegetarian. Her medical and psychiatric history was unremarkable. All other family members were fine. Her family history did not support the diagnosis.

She was initially evaluated in another tertiary care center where the diagnosis of major depressive disorder was made and sertraline was started. However, her condition gradually worsened. She started having difficulties remembering the names of family members, remembering whether she ate or not, performing simple tasks such as cooking, bathing, taking finances, etc, as well as difficulties with the names of common objects. This was followed by frequent episodes of visual hallucinations and catatonic stupor for several weeks. She also began to develop multiple myoclonic seizures along with akinetic mutism. She was diagnosed with major depression with psychosis and; therefore, she turned to our center for electroconvulsive therapy (ECT) and further treatment.

On examination, the vital signs were stable. The Glasgow Coma Scale was E4V2M3, and pupils were bilaterally equal and reactive. The fundus examination was normal. She had no signs of lymphadenopathy, meningism, glossitis, or dermatitis. Palmomental reflex was present on the left side while other frontal release signs were absent. Plantar reflexes were downgoing bilaterally. Muscle tone was increased in all four extremities. Bilateral biceps, triceps, and knee reflexes were 3+. No bruit was heard over the skull. The rest of the examinations revealed normal findings. We did not notice signs of primary tumor elsewhere in the body. With the provisional diagnosis of major depression with psychosis, she was admitted for ECT.

A complete blood count, hemoglobin, erythrocytic sedimentation rate, coagulation profile, liver, and renal function tests, C‐reactive protein, serum electrolytes (Na^+^, K^+^, Ca^2+^and Mg^2+^), serum glucose, and urinalysis were within normal limits. Chest X‐ray and Mantoux tests were normal. Antinuclear antibody, anti‐N‐Methyl‐D‐Aspartate receptor antibody, and anti‐Japanese Encephalitis IgM antibody were found to be negative. Cerebrospinal fluid (CSF) parameters were normal and adenosine deaminase in CSF was within a normal range. CSF culture revealed no growth of microorganisms. Opening pressure during lumbar puncture was not raised.

**Figure 1:**
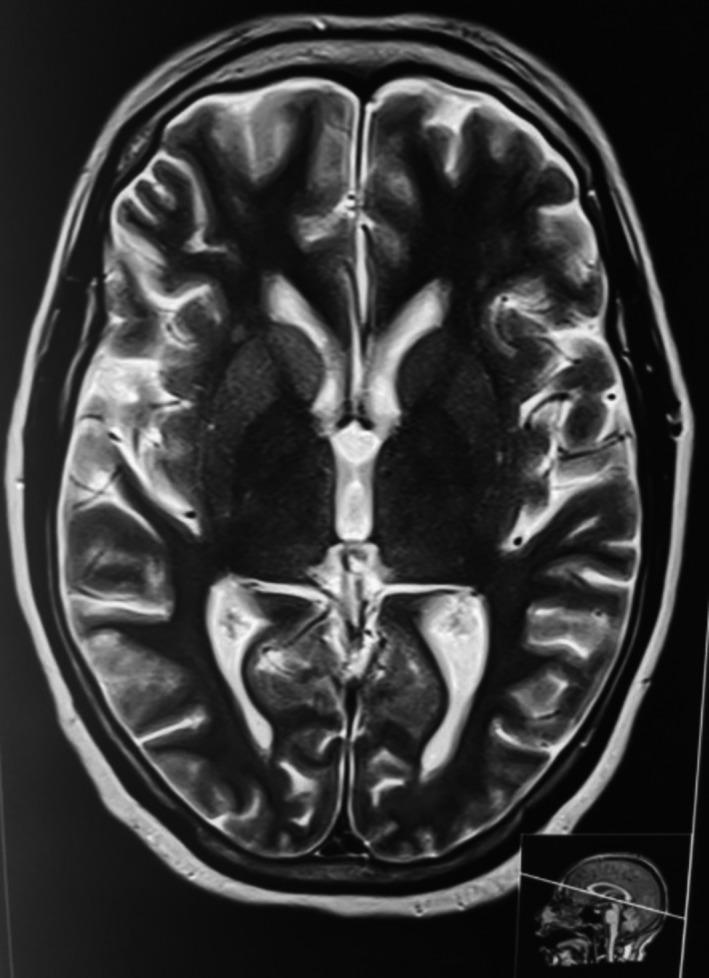
T2‐weighted MRI showing hyperintensity on right caudate and right lentiform nucleus

**Figure 2:**
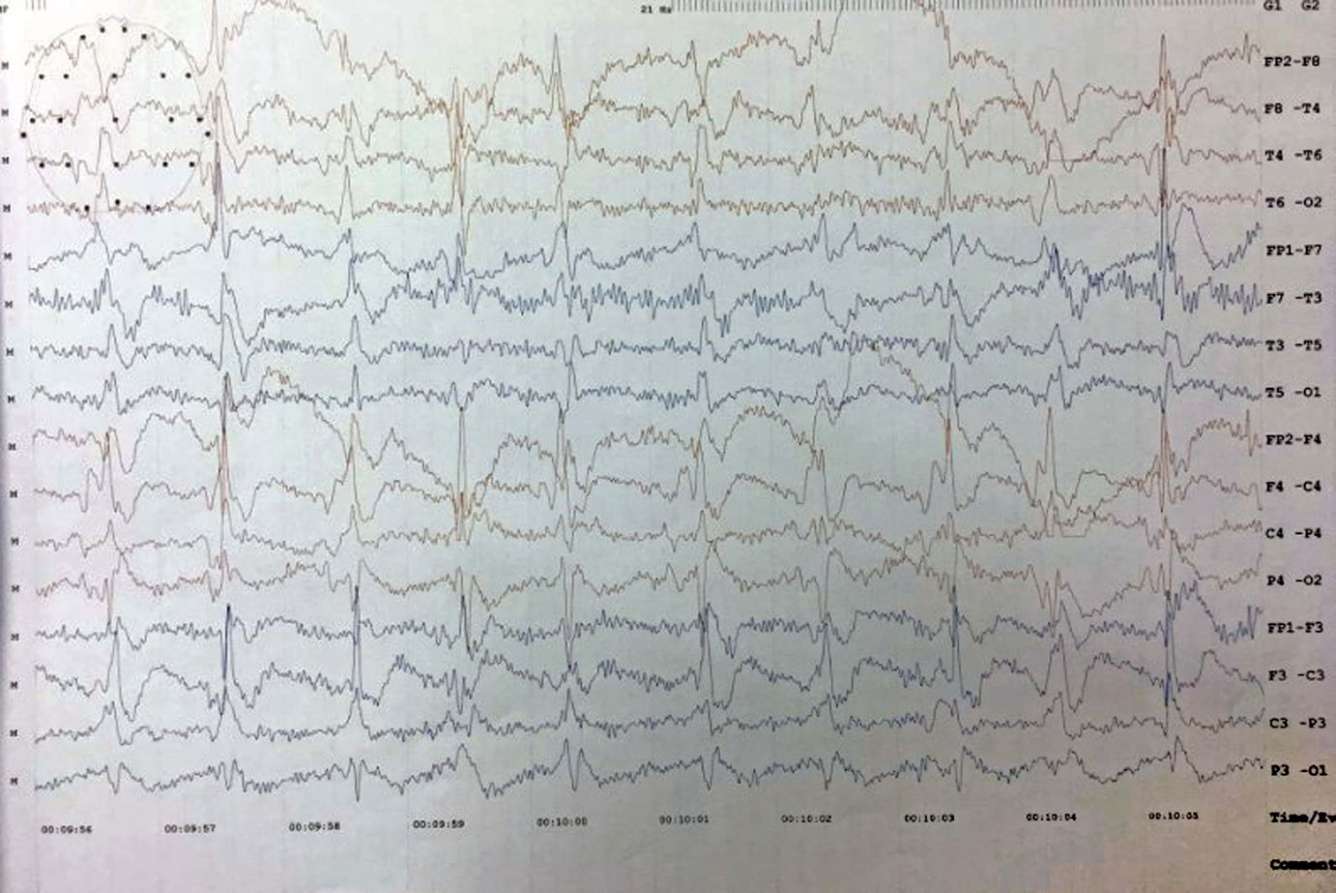
EEG showing background activity of diffuse, generalized, symmetric, synchronized high‐voltage sharp waves occurring periodically at a rate of 1 Hz with the electrode, movement, and muscle artifacts

T2‐weighted magnetic resonance imaging (MRI) showed increased intensity in the right caudate nucleus and right lentiform nucleus (Figure 1). The electroencephalogram (EEG) showed periodic biphasic discharge at 1 Hz (Figure 2). Investigation of CSF 14‐3‐3 and other markers of encephalitis was not done due to financial problems.

The diagnosis of probable sCJD was made. Supportive care with antiepileptics and antidepressants was provided. The patient's condition progressively worsened and she passed away due to aspiration pneumonia 33 days after admission.

## 3 DISCUSSION

Sporadic CJD is usually characterized by rapidly progressing dementia, ataxia, myoclonic seizures, or personality changes. Ocular symptoms can be present in the form of cortical blindness, distortion of visible objects, and paralysis of convergence or gaze. However, psychiatric manifestations, including depression, psychosis, and sleep disturbances, maybe the only manifestations of sCJD and thus are misdiagnosed. They later get worsened despite standard psychiatric treatments.^4^, ^5^, ^6^ As seen in our case, the accompanying cognitive deficits become obscured by prominent behavior and personality changes due to which a primary psychiatric disorder may be suspected.^7^

Usually, there is a rapid neuropsychiatric deterioration and death occurs within a year of symptom onset.^8^ In the same line, Josephs et al^9^ suggested that rapidly progressive dementia beyond 12 months duration is more likely to be a non‐CJD neurodegenerative disease. Our case had an acute onset of symptoms which was rapidly progressive until her demise at 3 months.

Magnetic resonance imaging is the imaging modality of choice in CJD. The classic MRI findings in sCJD are a high signal in the basal ganglia or cerebral cortex on diffusion‐weighted imaging (DWI) and fluid‐attenuated inversion recovery (FLAIR) sequences.^10^ Though MRI has high sensitivity and specificity for sCJD (96% and 93%, respectively),^11^ these findings are often confused with a stroke, vasculitis, or reversible posterior leukoencephalopathy.^12^, ^13^ Patients suspected with sCJD with high T2 signal intensity in basal ganglia were found to present with an earlier rapidly progressive dementia and shorter survival time (median 6.7 and 8.6 months, respectively) consistent with our case.^14^

The typical EEG appearances in sCJD are periodic, biphasic, or triphasic sharp‐wave complexes of 1‐2 Hz. These features present late in the disease process.^10^, ^15^ This is not usually seen in variant CJD. Periodic sharp‐wave complexes (PSWCs) are not only sensitive (64%‐66%) and specific (74%‐91%) diagnostic indicators in sCJD but also appropriate to differentiate probable sCJD from other prion diseases.^16^, ^17^ However, PSWCs were also recorded in Alzheimer's disease, vascular dementia, and dementia with Lewy body.^17^, ^18^ Therefore, neuroimaging is helpful in distinguishing prion‐related from nonprion‐related rapidly progressing dementia^19^ and is the best predictor for sCJD.^20^ Our case had a typical MRI sign in addition to PSWCs at EEG.

Routine CSF analysis for cell count, protein, and oligoclonal bands (usually positive in steroid‐responsive encephalitis associated with autoimmune thyroiditis (SREAT), autoimmune encephalitis, viral encephalitis, and multiple sclerosis) is normal in CJD.^21^, ^22^, ^23^ This was consistent in our case as well.

Because of its variable presentation, the diagnosis of CJD is challenging.^24^ Diagnostic criteria have been defined to identify this rare disorder. CDC's diagnostic criteria for CJD, 2018^25^ for probable sCJD require a history of progressive neurological syndrome with a positive real‐time quaking‐induced conversion (RT‐QuIC) test OR rapidly progressive cognitive impairment; and at least two out of the following four clinical features: myoclonus, visual or cerebellar symptoms, pyramidal or extrapyramidal symptoms, or akinetic mutism; and at least a positive finding on EEG (generalized periodic complexes), MRI or CSF (positive 14‐3‐3 protein) and without routine investigations indicating an alternative diagnosis.

Cerebrospinal Fluid biomarkers like 14‐3‐3, S100 beta, neuron‐specific enolase, and total tau have a controversial role in the diagnosis of sCJD as they are not prion specific proteins and have varying sensitivity and specificity. They are considered to be the markers of neuronal injury.^26^ A new diagnostic modality known as RT‐QuIC has remarkably high sensitivity and specificity in recent studies (sensitivity of 85.7% and specificity of 100%) in sCJD.^27^

Before proceeding with the diagnosis, we ruled out many other vascular/ischemic, infectious, toxic/metabolic, autoimmune, metastatic/neoplasm related, iatrogenic, systemic/seizures/sarcoid, and demyelinating causes of rapidly progressive dementia,^28^ on the basis of clinical presentation and best utilizing some investigations available in our setting.

Occasionally, some neurodegenerative diseases like rapidly progressive Alzheimer's dementia (AD), vascular dementia, dementia with Lewy bodies, frontotemporal dementia, chronic sinus thrombosis, and corticobasal degeneration follow a more rapidly progressive course than is typical and are misdiagnosed as sCJD.^18^, ^29^, ^30^ However, there was no extrapyramidal sign as might be noticed in AD. Accelerated weight loss, disturbed sleep pattern thought to indicate AD was not seen in our patient.^31^

Autoimmune encephalitis most commonly presents with early psychiatric manifestations and cognitive decline.^32^ Hashimoto's encephalopathy may have a relapsing‐remitting course with stroke‐like symptoms whereas the course of sCJD is fulminant, leading to death within 1 years' time in 85% of patients.^19^

Symptoms typically present in vascular pathologies like headache and localizing motor, visual or sensory signs were absent in our case. She had no risk factors for vascular diseases. Cognition was not suddenly impaired as might be seen in strategic infarcts.^24^ Her coagulation profile was normal as well.

Comparing with the clinical features in the diagnostic criteria (mentioned above), our patient had a rapidly progressive cognitive impairment, myoclonus, and akinetic mutism. EEG and T2‐weighted MRI were characteristic for confirming the clinical diagnosis of CJD. Thus, our case should finally be classified as probable sCJD. The presence of akinetic mutism together with the classical EEG suggests that she presented to us in the late stage of the disease process. We did not test for CSF 14‐3‐3 protein and genetic mutations due to the financial problems of the patient. Though the family history was unremarkable, still the possibility of a genetic form of CJD could not be definitely eliminated.

## 4 CONCLUSION

A definitive diagnosis of CJD is possible only through histological examination of the brain. The diagnosis is challenging in resource‐limited settings. It is important to consider CJD in elderly patients presenting with rapidly worsening depression unresponsive to antidepressants. This invariably fatal condition has no treatment other than symptomatic management and palliative care. All aspects of this rare disease require further research.

## CONFLICT OF INTEREST

The author(s) declare(s) that there is no conflict of interest regarding the publication of this paper.

## AUTHOR CONTRIBUTIONS

HK, PA, and NBP: wrote the initial draft of the manuscript. NBP, ZK, and GN: edited the draft and reshaped it into this manuscript. All authors approved the final version of the manuscript and agree to be accountable for all aspects of the work in ensuring that questions related to the accuracy or integrity of any part of the work are appropriately investigated and resolved.

## ETHICAL APPROVAL

Need for ethical approval waived. Consent from the patient's daughter deemed to be enough.

## CONSENT FOR PUBLICATION

Written informed consent was obtained from the patient's daughter for publication of this case report and any accompanying images. A copy of the written consent is available for review by the editor‐in‐chief of this journal.
